# A Systematic Review of Normative Studies Using Images of Common Objects

**DOI:** 10.3389/fpsyg.2020.573314

**Published:** 2020-12-04

**Authors:** Cristiane Souza, Margarida V. Garrido, Joana C. Carmo

**Affiliations:** ^1^Iscte-Instituto Universitário de Lisboa, Cis-IUL, Lisbon, Portugal; ^2^Faculdade de Psicologia, Universidade de Lisboa, Lisbon, Portugal

**Keywords:** norms, images of common objects, semantic, perceptive, affective

## Abstract

Common objects comprise living and non-living things people interact with in their daily-lives. Images depicting common objects are extensively used in different fields of research and intervention, such as linguistics, psychology, and education. Nevertheless, their adequate use requires the consideration of several factors (e.g., item-differences, cultural-context and confounding correlated variables), and careful validation procedures. The current study presents a systematic review of the available published norms for images of common objects. A systematic search using PRISMA guidelines indicated that despite their extensive use, the production of norms for such stimuli with adult populations is quite limited (*N* = 55), particularly for more ecological images, such as photos (*N* = 14). Among the several dimensions in which the items were assessed, the most commonly referred in our sample were familiarity, visual complexity and name agreement, illustrating some consistency across the reported dimensions while also indicating the limited examination of other potentially relevant dimensions for image processing. The lack of normative studies simultaneously examining affective, perceptive and semantic dimensions was also documented. The number of such normative studies has been increasing in the last years and published in relevant peer-reviewed journals. Moreover, their datasets and norms have been complying with current open science practices. Nevertheless, they are still scarcely cited and replicated in different linguistic and cultural contexts. The current study brings important theoretical contributions by characterizing images of common objects stimuli and their culturally-based norms while highlighting several important features that are likely to be relevant for future stimuli selection and evaluative procedures. The systematic scrutiny of these normative studies is likely to stimulate the production of new, robust and contextually-relevant normative datasets and to provide tools for enhancing the quality of future research and intervention.

## Introduction

Objects constitute a distinctive type of stimuli that entail specific visual processing as compared, for example, to faces or words (e.g., Tanaka and Taylor, 1991; Farah, 1992, 2004). Images of objects are frequently used in research and interventional practices, particularly those objects that are commonly encountered in everyday-life (e.g., Palmer, 1975; Treisman, 1986; Farah, 1992; Kouststaal et al., 2003; Reber et al., 2004; Brady et al., 2008; Souza et al., 2016). Common objects comprise concrete and depictable items from living things (e.g., a “cat” for “Mammals”) and non-living things (e.g., a “car” for “Vehicles”) (see Capitani et al., 2003; Borghi et al., 2017). They differ from other types of objects (e.g., novel, artificial or abstract, see Kouststaal et al., 2003 for an example) especially regarding the type of conceptual knowledge associated to them. According to objects categorization frameworks, common objects are linked to categories from distinct levels of abstraction, from high (e.g., “Vehicles”) to low (e.g., “City bus”) with basic categories (e.g., “car”) being the most inclusive ones (since their members share more conceptual, motor, and/or perceptual attributes/characteristics) and often presenting an advantage in learning, classification and retrieval (Rosch et al., 1976; Tanaka and Taylor, 1991). Thus, because they are meaningful, common objects involve associated general knowledge that is recurrently present in our daily-life experiences (i.e., learning, talking, cooking, identifying/finding objects, etc.), and that is highly relevant when trying to understand and interact with the world. These particular characteristics of common objects make them extremely useful for affective and neurocognitive tasks that require participants to recognize items and to make categorical decisions about them (e.g., VanRullen and Thorpe, 2001; Konkle et al., 2010).

However, images of objects may vary in several dimensions, such as surface details, the categories, and even the cultural background of the perceivers. In addition to their associated semantic knowledge, the mental representation of such visual items can include several item-attributes, namely perceptual features (contrast, color, multi-D shape, reflectance, luminance, moving, and orientation), contextual occurrence and also the emotions they elicit (see Palmer, 1975; Treisman, 1986; Bonin et al., 2003; Brady et al., 2008). For instance, different exemplars of the same object (e.g., different types of cats or different exemplars of cars) have distinct perceptual characteristics (e.g., different colors, luminance, viewpoint or distinct shapes). For example, a specific exemplar of a given category may be more frequent in one culture than in other (e.g., Peterbald cats are more frequent in Russia and a Tuk-Tuk vehicle is common in India but not in England or Brazil) or may be differently processed according to the categorization context (i.e., a boot may be considered as clothes or as work equipment depending on their function). Therefore, their visual representation combines surface features with our predictive capabilities (expectancy) derived from our previous experience (that are both meaningful and emotional). In fact, a recent Bayesian meta-analytic study about picture-name norms of line-drawings of objects indicated that several subjective dimensions, namely image agreement, name agreement, familiarity, imageability and age-of-acquisition, constitute strong predictors of picture-naming abilities that may influence pre or pos lexical processing (Perret and Bonin, 2019). Moreover, differences regarding the cultural background and linguistic variations also provided intriguing outputs (Dell'Acqua et al., 2000; Boukadi et al., 2016; Duñabeitia et al., 2018). These findings converge in suggesting that the same image of an object can be processed differently depending upon many aspects.

The widespread use of images of common objects in research and intervention must acknowledge the high variability of these items and their related properties, which require careful selection procedures and control for the possible influence of several dimensions potentially co-occurring during the manipulations of interest. This can only be ensured through careful standardization procedures. Normative studies have become increasingly more sophisticated and innovative, integrating theoretical and methodological knowledge from several other areas, such as Psychometrics, Computer Sciences, Neuroscience, Psycholinguistics, or Visual processing. However, review studies may also constitute valuable guidelines in selecting relevant datasets, clarifying standardization methodologies and identifying factors to be controlled (Perret and Bonin, 2019). In the current review, we critically summarize the main features of normative studies using images of common objects, with particular emphasis on the stimuli dataset characteristics and standardization procedures.

### Why Is It Important to Normalize Images of Common Objects?

Images of common objects are frequently used as stimulus materials because such items are easily and generally accessed and understandable. Furthermore, there are specific research and intervention areas, such as linguistics, developmental neuropsychology, and cognitive neuroscience, in which such images are particularly useful and required. For example, images of common objects are extensively used in the examination of naming abilities and in memory research (e.g., Semenza, 2009; Kavé et al., 2018), in the examination of neurocognitive performance related to categorical processing (e.g., Martin et al., 1996), in visual perception studies with well-known items (e.g., Brady et al., 2009), and also in emotional processing research (e.g., Kensinger and Schacter, 2006).

However, the use of visual stimuli in research requires the careful examination of their image properties and how they can impact several mental functions (see Snodgrass and Vanderwart, 1980). Image attributes (color patterns, valence, familiarity, etc.) are known to influence performance in several cognitive tasks (Taylor et al., 1998; Ullman et al., 2002; Holmes and Ellis, 2006; Mendonça et al., 2020). For example, several studies have shown the facilitating effect of perceptual details (color, shape, brightness, visual complexity) in object naming, categorization and recognition (see Price and Humphreys, 1989; Ullman et al., 2002, for more details). Likewise, affective dimensions, such as arousal and valence were shown to modulate cognitive processes, such as memory and semantic judgment (Kensinger and Schacter, 2006; Kensinger, 2007). The influence of semantic variables on the processing of these items was also evidenced by the effects of different categories and their distinct domains (Warrington and Shallice, 1984; Moss and Tyler, 1997) as well as by the influence of typicality in object processing (Holmes and Ellis, 2006). It has also been shown that different types of stimuli require their normalization in different dimensions (see Prada et al., 2010, 2017; Garrido et al., 2016) that may enhance their applicability. For example, meaningfulness is important for symbols' processing but not so much for facial stimuli. Likewise, distinctiveness might be more relevant for processing uncommon-discriminative items, such as unlikely events, landmarks, or people's faces in comparison with images of common objects.

The standardization of images of common objects assumes particular relevance since they are usual, frequent and expected in everyday life and recurrently used in scientific studies. This was long acknowledged in the classic work by Snodgrass and Vanderwart (1980) that constitutes a landmark in the production and normalization of visual databases of common objects for research purposes. The authors argued that visual material (alike verbal items) should also be standardized to avoid potential biases in research. Based on a sample of 219 English-speaking graduate students, they provided norms for 260 black-and-white line-drawing illustrations of common objects regarding naming and familiarity for the semantic domain as well as visual complexity and image agreement for the perceptive domain. The relevance of their findings rests on the identification of subjective independent attributes of images that potentially influence several cognitive tasks—like free-recall, go-no go and emotional processing tasks—and constituted an important step toward a proper validation of visual stimuli. Moreover, Snodgrass and Vanderwart work 1980 was critical for emphasizing the importance of conducting normative studies with images.

The work of Snodgrass and Vanderwart (1980) has been subsequently extended, across different age samples (Berman et al., 1989; Yoon et al., 2004), distinct cultures (Alario and Ferrand, 1999; Pind et al., 2000; Pompéia et al., 2001; Nishimoto et al., 2005; George and Mathuranath, 2007; Manoiloff et al., 2010; Boukadi et al., 2016), with increased variety of visual stimuli (Cycowicz et al., 1997; Morrison et al., 1997) and with refined parameters (e.g., surface details and texturized or colorized stimuli in Rossion and Pourtois, 2004). The repeated and consistent application of the Snodgrass and Vanderwart (1980) database has turned it into a well-established image dataset that constitutes a main reference in the field, as well as an important resource for researchers and other professionals. However, aside from these pictographic studies, other databases of images of common objects seem to have been poorly widespread despite of their great scientific relevance.

### Why Is It Relevant to Summarize the Development of Standardization Practices?

The selection of stimuli constitutes an important step during the planning of experimental studies (see Snodgrass and Vanderwart, 1980; Brodeur et al., 2014). Moreover, the use of previously standardized stimulus should permit the comparison between different studies and allow the reproduction of the materials and methods across research teams, as requested for replicability purposes (Wilcox and Claus, 2017). In contrast, an inconsistent use of the stimuli across studies in a given research field along with the lack of careful stimuli standardization procedures makes any comparison between outputs unfeasible or, at best, little informative. Recently, researchers have been increasingly concerned about the quality of the visual stimuli used in their studies and the knowledge of their properties (with more than 200 normative studies published between 1996 and 2016). As highlighted by Brodeur et al. (2014), normative studies have been crucial to increase the adjustment of the stimuli to the research purposes, allowing a more precise characterization of the stimuli, the control of confounding effects as well as a better manipulation of the variables of interest. In addition, the establishment of norms also provides important insights about the items processing and their cultural appropriateness (Brodeur et al., 2012; Prada et al., 2016).

Nevertheless, the careful standardization procedures required for using such images are not always conducted, once they imply time, knowledge, and resources. Standardization involves stimuli construction and selection, as well as extensive data collection and analysis. Additionally, it may also require expertise in specific metrics (e.g., computational models for surface features, h-index of naming, mediation models, item characteristics curves, etc.) as well as cross-country evaluations that consider the influence of language variations and cultural specificities. Moreover, the norms already produced are not always available, and even when they are, the stimuli selection must often be adapted to the researchers' goals and to the specific cultural contexts. However, the current demands of scientific practice and the pressure for publication often conflict with the time-consuming steps prior to experimental studies. As a consequence, the control for stimuli diversity and related confounding factors established in previous normative procedures are often misinterpreted as an obstacle instead of a step toward increasing quality in research.

In sum, the process of producing and selecting stimuli constitutes an important but also complex and costly task. Therefore, a systematic review of the standardization studies of interest might be highly relevant in assisting this first and essential phase of planning the research in order to identify, access and select adequate stimuli and potentially relevant dimensions. Given its particularities and its widespread use, it is crucial to systematically examine the available normative studies using image datasets of common objects to uncover their specificities, their standardization practices as well as the potential gaps in those studies. Finally, the systematic information about which normative studies have been produced for common objects constitutes a valuable resource for electing adequate procedures and databases as well as acknowledge common objects as a relevant general category.

### The Current Study

The systematized knowledge from these normative studies, constitutes an important resource regarding the stimuli characteristics but also a valuable asset to identify well-established practices.

A systematic review of the literature was conducted using the PRISMA (Preferred Reporting Items for Systematic reviews and Meta-Analyses; see Liberati et al., 2009, for details about this methodological procedure) on standardized norms for images of common objects obtained with adult populations in order to establish the current state of the art in research on normative studies using images of common objects (see PICOS format in our online protocol at https://protocols.io/view/a-systematic-review-of-normative-studies-using-ima-bbysipwe [doi: 10.17504/protocols.io.bbysipwe]) Specifically, the present systematic review aimed to:

(1) Identify and characterize the published normative studies of images of common objects with adults, in order to assist the selection and further production of such stimuli and new databases [How many normalized datasets are available in peer-reviewed literature? What are their sources (i.e., journals, journals h-index, temporal distribution)? What are their general characteristics (sample characteristics, type of stimuli, stimuli production procedures, linguistic and cultural-based examinations)? Are these studies using different categories of common objects? If so, are they addressing the effects of category and domain?]
(2) Determine and critically examine the most reported evaluative dimensions and their parameters in common objects normative studies, to uncover the most relevant properties to examine in normalizing images of common objects [Which are the main dimensions reported, their scales and task instructions? Is there consistency in the evaluated dimensions and their parameters?]
(3) Critically appraise the reliability of the norms produced, without losing their cultural specificity, by inspecting the coherence of ratings and their correlations reported across normative studies of images of common objects [How are images of common objects rated across studies? How are the main evaluative dimensions correlated across studies?]
(4) Uncover the application potential of each elected normative study by an exploration of the availability of the databases (i.e., whether the dataset and their norms are publicly available) and their impact (i.e., citation score) as potentially relevant indicators for selecting, producing or replicating normative studies [How accessible and widespread these databases are (availability and impact)?].

## Methods

### Protocol, Search Strategy, and Eligibility Criteria

The systematic review conducted followed the PRISMA guidelines (Liberati et al., 2009). Further details on previously defined methodological guidelines are available in our protocol page (https://protocols.io/view/a-systematic-review-of-normative-studies-using-ima-bbysipwe [10.17504/protocols.io.bbysipwe]) The PRISMA checklist is included as Supplementary Material S1.

The search strategy included a first stage of systematic electronic search in online sources to identify the relevant normative studies published in English in academic peer-reviewed journals. Four databases were explored in the EBSCOhost platform to find potentially relevant studies: Academic Search Complete (1976–2019), PsycINFO (1948–2019), Psychology and Behavioral Science (1950–2019), and PsycARTICLES (1948–2019). The search terms entered in a Boolean phrase search mode using all possible combinations were the following: (a) validation OR norms; AND (b) pictures OR images; AND (c) typicality OR familiarity OR name-agreement OR valence OR arousal OR aesthetic OR “visual complexity” OR categories; (d) NOT social OR body parts OR face OR emotion^*^ OR ^*^MRI OR neuroimaging. The search terms used in (c) were based on dimensions commonly reported in the literature. The (d) search entries were included to filter an extensive list of articles that refer to the words “image,” “picture,” and “norms.” The search was conducted without year restrictions or entry boundaries (title, subjects/keywords, or abstract). An additional search was conducted on Scopus and on Web of Science databases with the same Boolean criteria and without year parameter, defining as search criteria: type of document “article” and “English” language. On Scopus, the search was conducted in title, abstract, and keywords. On WoS, the search was limited to the title. The analysis of overlapping articles and the management of the selected articles was made using EndNote X8 software. A complementary hand search phase was also conducted based on known authors/papers including pertinent normative studies using images of common objects not captured by the automatic search. The search procedures and the collection of the articles were completed by June, 2019.

The inclusion criteria for electing potential studies involved three cumulative conditions: (1) the inclusion of healthy adult participants (minimum of 18 years-old); (2) the standardization of images of common objects into categories of the living and/or non-living domains (not social or emotional representations, not action scenes, not objects in context, not human images); (3) at least one the following dimensions as independent variable: semantic dimensions (i.e., name-agreement, category-agreement, familiarity, typicality), affective dimensions (i.e., aesthetic appeal, arousal, valence) and perceptive dimensions (i.e., visual complexity, picture-name agreement).^1^ On EBSCOhost platform, the age restriction (≥18 years old) was introduced during the online search. The inclusion criteria (image type and dimensions) were also confirmed during a subsequent inspection of the data by the title and the abstract to select the relevant studies.

### Selection of Studies, Risk of Bias, and Data Treatment

The second stage of the search (see PRISMA guidelines) involved the screening of the data by title, abstract, and full text, by three independent judges using the Rayyan QCRI (Qatar Computing Research Institute, Hamad Bin Khalifa University) web application. The selection by title and abstract intended to control for subjective bias in the selection of the articles as well as to efficiently filter the relevant articles, confirming the inclusion criteria. With this procedure, articles from the previously comprehensive search that did not include image validation studies (e.g., validation of instruments, self-image studies) or studies that were not pertinent to the current review (e.g., images of emotional expressions of disgust or fear, parts of the body, objects in a scenery, pairs of objects) were excluded. Subsequently, the full-text examination ensured the eligibility of the selected studies to be retained based on all inclusion criteria. Disagreements on retaining or excluding on each screening phase were discussed until a consensus was reached.

To our knowledge there is no specific standardized tool available for quality assessment in normative studies. In the present research the Cochrane Collaboration Risk of Bias tool (Higgins and Altman, 2008) was used as a consistent parameter in quality assessment. Our goal was to provide a broad and comprehensive analysis of the methodological procedures. However, excluding articles by methodological reasons could constitute a bias. Therefore, the quality assessment is provided in a qualitative manner and is merely informative instead of a requisite for maintaining an article in the sample (see Supplementary Material S3).

Data extraction was performed using a qualitative systematization of the relevant information for answering the previously defined research questions. Two coders extracted and systematized information from each study included to complete a previously established resume-table. When appropriate, complementary variables were included to guarantee the specificity of the information (e.g., for the type of categories, the “category name” as well as the “semantic level type” were extracted). The extracted information included: (a) bibliometric information and indicators (journals, journals h-index, temporal distribution); (b) general characteristics and standardization practices of images of common objects (sample characteristics, language and cultural variations, procedures, stimulus characteristics); (c) dimensions reported in the standardization of images of common objects (main dimensions reported, scales and task instructions, and their consistency across studies); (d) assessment of images of common objects (mean ratings and correlational results, reliability of the datasets); (e) accessibility and application potential of the normative databases (availability and citation impact).

For the general characteristics of the studies, sample characteristics comprised N of participants, mean age, age range, schooling, schooling range; language and cultural variations included language/cultural context and cross-cultural comparisons; procedures entailed data collection procedure; and stimulus characteristics discriminated if it was S&V stimuli replication/adaptation/extension, the stimuli description, stimuli type, image resolution, number of stimuli, number of stimuli/participant, categories of the items—number and types. Moreover, the cross-cultural comparisons described the presence of comparisons, the type of comparison (if between or within studies), the sample source (if they compared the same database or not) and the statistical methods used for cross-cultural analysis (i.e., correlations, multiple regression, *t*-test, ANOVA). In (c), a qualitative appraisal of item norms was provided by examining the dimensions reported (evaluated dimensions; instructions and scales). Main findings for the most reported dimensions were also included in (d), namely assessment of images (mean ratings by study; correlational results by study—*r* and *p*-values) of all studies reporting overall results for images of common objects. Correlational results for each dimension between studies were also considered for the main dimensions. Finally, information about the impact (i.e., number of citations) and availability (i.e., whether the database and their norms are freely available) of the database was also collected (e). Such indicators are predictors of the scientific impact of the articles in their respective areas (i.e., applicability) and also reflect their potential for replication. The overall findings were summarized in qualitative (i.e., descriptive) and quantitative (i.e., frequencies and percentages) tables and figures.

### Retrieval of the Studies and Literature Selection

**Figure 1:**
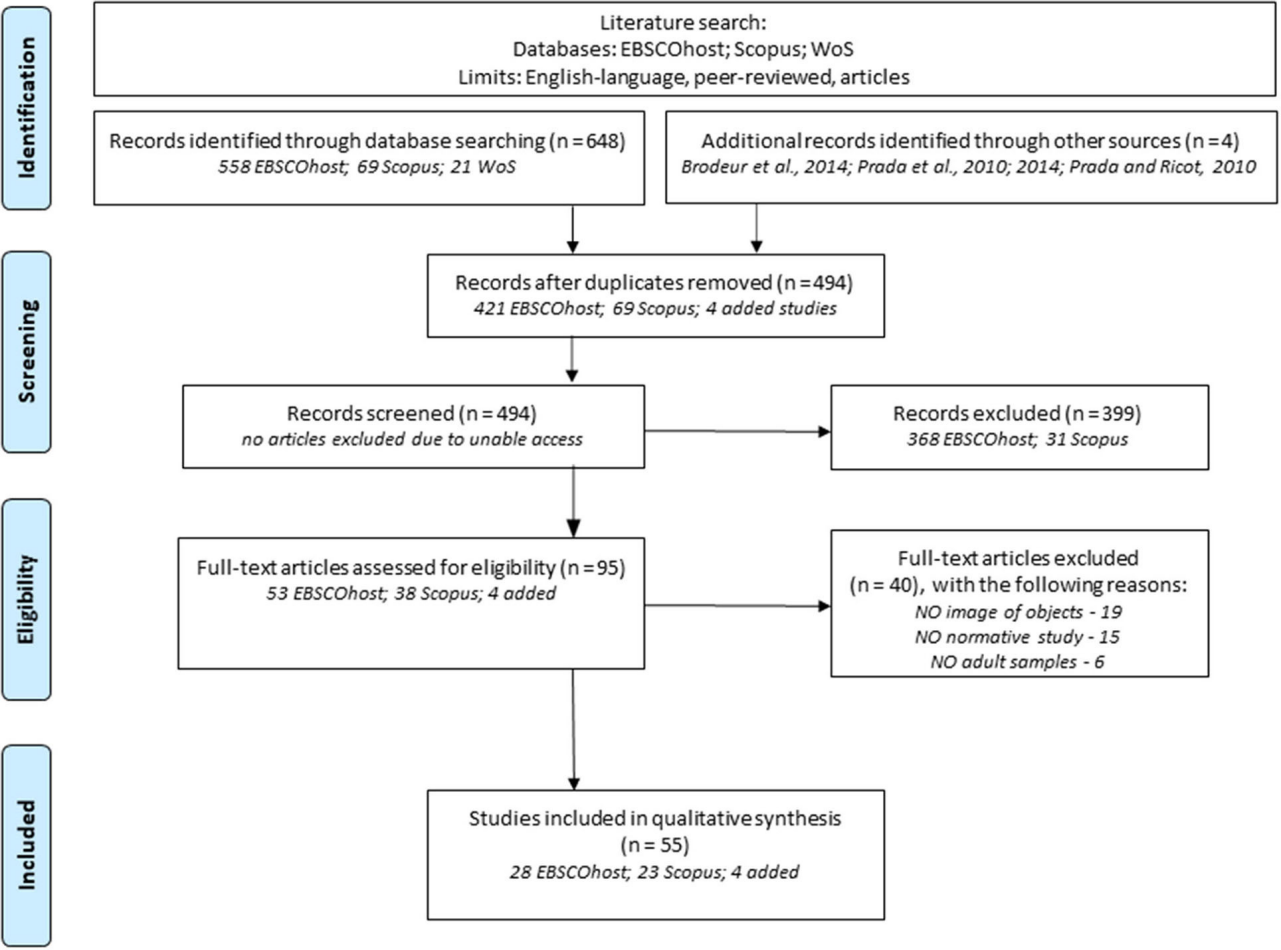
Flow diagram of studies' selection process, using the PRISMA method (adapted from Liberati et al., 2009).

The first stage of the systematic electronic search produced a combined result of 648 articles: 558 from the EBSCOhost database (334 from Academic Search Complete, 187 from PsycINFO, 25 from Psychology and Behavioral Sciences Collection and 12 from PsycARTICLES), 69 from Scopus and 21 from WoS. Four additional relevant studies were inserted on the data (i.e., Prada and Ricot, 2010; Prada et al., 2010, 2014; Brodeur et al., 2014) during the hand search phase. Despite not meeting all the inclusion criteria (some were not written in English) or not being retrieved in the systematic search (i.e., Brodeur et al., 2014), the inclusion of these articles was justified by their reporting of affective dimensions that were not explored in the elected papers (i.e., Prada and Ricot, 2010; valence and arousal examined in Prada et al., 2010) or because of the high number of images of common objects included (Brodeur et al., 2014). After removing duplicates, the number of articles to be screened was reduced to 494 (see PRISMA flow diagram in Figure 1). The results from screening by title and abstract lead to the retention of 95 (53 from EBSCOhost, 38 from Scopus, and 4 hand-search) and the elimination of 368 articles from EBSCOhost and 31 from Scopus. Finally, a full-text analysis of the 95 articles, lead to the exclusion of 40 (see Supplementary Material S2, for excluded articles list) and narrowed the sample to 55 full-text elected articles (28 from EBSCOhost, 23 from Scopus, and 4 from the hand-search) for a qualitative synthesis. The exclusion of articles was motivated by the following reasons: different stimuli type (faces, body parts, neural image, sounds, words, action pictures, food images, etc.); not normative study (e.g., literature review or correlational studies); incongruent theme (e.g., neural network, pelvis fracture images); samples (e.g., children or clinical).

## Results

The qualitative appraisal of the retained studies focused on the identification and categorization of their characteristics that were relevant to our aims. Overall, the final sample included a reasonable number of papers (*n* = 55) presenting norms for images of common objects grouped in two distinct types of visual representations: line-drawings (*n* = 39, 70.9%), photographs (*n* = 14, 25.5%), or both (*n* = 2, 3.6%). The analysis of the publications examining the different stimuli type (i.e., line-drawings, photos or both) across the years (i.e., older articles—up to 2009 vs. recent articles—from 2010 to 2019) revealed an earlier trend for publishing datasets using drawings (line-drawings: 95.8%; photos: 4.2%) and an increased interest in ecological stimuli or its comparison with drawings in recent years (line-drawings: 51.6%; photos: 41.9%; both: 6.5%). The following subsections present the systematization of the information available in the studies sample considering the previously reported categories of data treatment. For each of these categories, the results of normative studies using photographs are emphasized because of their relevance in introducing ecological validity and the documented increased interest in this type of stimuli. A descriptive summary of the results is presented in Table 1. A qualitative summary of the main reported dimensions (with their instructions, scales, and instruction focus) is also provided in Table 2. Supplementary tables with all the data extracted and the distribution of quantitative norms results (i.e., item norms by dimension) for each relevant dimension and their correlations in each study is also provided in Supplementary Materials S3, S4, respectively.

**Table 1:** Summary table of the main characteristics of the studies sample by Stimuli Type (absolute frequencies and percentage).

	**Overall**		**Stimuli Type**					
	**(*****n*** **= 55)**		**Line-drawing (*****n*** **= 39)**		**Photographs (*****n*** **= 14)**		**Both (*****n*** **= 2)**	
	***N***	***%***	***n***	***%***	***n***	***%***	***N***	***%***
**AGE**								
Young adults	30	54.5	21	53.8	8	57.1	1	50.0
Mid-age adults	10	18.2	6	15.4	4	28.6	0	0.0
Older adults	1	1.8	1	2.6	0	0.0	0	0.0
Multiage	14	25.5	11	28.2	2	14.3	1	50.0
**SCHOOLING LEVEL**								
High school	1	1.8	1	2.6	0	0.0	0	0.0
Undergraduate	36	65.5	27	69.2	7	50.0	2	100.0
Graduate/Post-graduate	1	1.8	1	2.6	0	0.0	0	0.0
Undergraduate and Graduate	5	9.1	4	10.3	1	7.1	0	0.0
n.d.	12	21.8	6	15.4	6	42.9	0	0.0
**LANGUAGE**								
English	14	25.5	9	23.1	3	21.4	2	100.0
French	6	10.9	5	12.8	1	7.1	0	0.0
Spanish	6	10.9	3	7.7	3	21.4	0	0.0
Portuguese	4	7.3	1	2.6	3	21.4	0	0.0
Italian	3	5.5	2	5.1	1	7.1	0	0.0
Turkish	2	3.6	2	5.1	0	0.0	0	0.0
Russian	2	3.6	2	5.1	0	0.0	0	0.0
Arabic	2	3.6	2	5.1	0	0.0	0	0.0
Chinese	2	3.6	1	2.6	1	7.1	0	0.0
Japanese	2	3.6	2	5.1	0	0.0	0	0.0
Dutch	2	3.6	1	2.6	1	7.1	0	0.0
Thai	1	1.8	0	0.0	1	7.1	0	0.0
Greek	1	1.8	1	2.6	0	0.0	0	0.0
Indian	1	1.8	1	2.6	0	0.0	0	0.0
Persian	1	1.8	1	2.6	0	0.0	0	0.0
Icelandic	1	1.8	1	2.6	0	0.0	0	0.0
Cross-linguistic	5	9.1	5	12.8	0	0.0	0	0.0
**DATA COLLECTION ENVIRONMENT**								
Experimental	25	45.5	15	38.5	10	71.4	0	0.0
Survey	18	32.7	13	33.3	4	28.6	1	50.0
Both	10	18.2	9	23.1	0	0.0	1	50.0
n.d.	2	3.6	2	5.1	0	0.0	0	0.0
**ONLINE RESOURCES**								
Yes	2	3.6	1	2.6	0	0.0	1	50.0
No	51	92.7	36	92.3	14	100.0	1	50.0
n.d.	2	3.6	2	5.1	0	0.0	0	0.0
**S&V (ORIGINAL, ADAPTATION, OR EXTENSION)**								
Yes	31	56.4	29	74.4	0	0.0	2	100.0
No	24	43.6	10	25.6	14	100.0	0	0.0
**STIMULI COLOR**								
Color	16	29.1	5	12.8	11	78.6	0	0.0
Black and white	35	63.6	32	82.1	3	21.4	0	0.0
Both	4	7.3	2	5.1	0	0.0	2	100.0
**STIMULI SIZE/RESOLUTION**								
Medium (up to 500px)	15	27.3	9	23.1	4	28.6	2	100.0
High (from 501px)	15	27.3	8	20.5	7	50.0	0	0.0
n.d.	25	45.5	22	56.4	3	21.4	0	0.0
**N OF STIMULI**								
Up to 50	1	1.8	0	0.0	1	7.1	0	0.0
51–100	5	9.1	4	10.3	1	7.1	0	0.0
101–200	5	9.1	3	7.7	2	14.3	0	0.0
200+	44	80.0	32	82.1	10	71.4	2	100.0
**N STIMULI/PARTICIPANT**								
Up to 50	4	7.3	2	5.1	2	14.3	0	0.0
51–100	5	9.1	4	10.3	1	7.1	0	0.0
101–200	9	16.4	4	10.3	5	35.7	0	0.0
200+	33	60.0	26	66.7	6	42.9	1	50.0
n.d.	4	7.3	3	7.7	0	0.0	1	50.0
**N CATEGORY**								
1–5	8	14.5	6	15.4	2	14.3	0	0.0
6–10	4	7.3	2	5.1	2	14.3	0	0.0
11–15	27	49.1	22	56.4	4	28.6	1	50.0
16+	7	12.7	1	2.6	5	35.7	1	50.0
n.d.	9	16.4	8	20.5	1	7.1	0	0.0
**CATEGORY-LEVEL**								
Basic level	10	18.2	4	10.3	6	42.9	0	0.0
Domain and basic level	2	3.6	2	5.1	0	0.0	0	0.0
Superordinate level	24	43.6	19	48.7	3	21.4	2	100.0
Basic and superordinate level	9	16.4	7	17.9	2	14.3	0	0.0
Domain. basic and superordinate level	1	1.8	0	0.0	1	7.1	0	0.0
n.d.	9	16.4	7	17.9	2	14.3	0	0.0
**CROSS-CULTURAL COMPARISON**								
Yes	34	61.8	27	69.2	6	42.9	1	50.0
No	21	38.2	12	30.8	8	57.1	1	50.0
**DATASET COMPARISON OF CROSS-CULTURAL COMPARISON**								
Direct	26	47.3	21	53.8	5	35.7	0	0.0
Indirect	4	7.3	3	7.7	0	0.0	1	50.0
Both	4	7.3	3	7.7	1	7.1	0	0.0
Absent	21	38.2	12	30.8	8	57.1	1	50.0
**Samples source of cross-cultural comparison**	(*n* = 34)		(*n* = 27)		(*n* = 6)		(*n* = 1)	
Between studies	29	85.3	22	81.5	6	100.0	1	100.0
Within studies	4	11.8	4	14.8	0	0.0	0	0.0
Both	1	2.9	1	3.7	0	0.0	0	0.0
**Statistical method for cross-cultural analysis**	(*n* = 34)		(*n* = 27)		(*n* = 6)		(*n* = 1)	
Correlations	24	70.6	19	70.4	5	83.3	0	0.0
Correlations and multiple regressions	3	8.8	3	11.1	0	0.0	0	0.0
ANOVAS/*T*-tests	4	11.8	3	11.1	0	0.0	1	100.0
ANOVAS/*T*-tests and Correlations/Regressions	3	8.8	2	7.4	1	16.7	0	0.0
**JOURNAL**								
Behavior Research Methods	27	49.1	24	61.5	2	14.3	1	50.0
PLoS ONE	4	7.3	0	0.0	3	21.4	1	50.0
Laboratório de Psicologia	3	5.5	0	0.0	3	21.4	0	0.0
Journal of Psycholinguistic Research	2	3.6	1	2.6	1	7.1	0	0.0
Journal of Clinical and Experimental Neuropsychology	2	3.6	1	2.6	1	7.1	0	0.0
Frontiers in Psychology	2	3.6	0	0.0	2	14.3	0	0.0
Quarterly Journal of Experimental Psychology	1	1.8	1	2.6	0	0.0	0	0.0
Quarterly Journal of Experimental Psychology Section A: Human Experimental Psychology	1	1.8	1	2.6	0	0.0	0	0.0
Applied Neuropsychology	1	1.8	1	2.6	0	0.0	0	0.0
Frontiers in Human Neuroscience	1	1.8	0	0.0	1	7.1	0	0.0
Annals of Indian Academy of Neurology	1	1.8	1	2.6	0	0.0	0	0.0
Brain and Cognition	1	1.8	1	2.6	0	0.0	0	0.0
Neurological Sciences	1	1.8	1	2.6	0	0.0	0	0.0
Aging. Neuropsychology. and Cognition	1	1.8	0	0.0	1	7.1	0	0.0
Journal of Experimental Child Psychology	1	1.8	1	2.6	0	0.0	0	0.0
Scandinavian Journal of Psychology	1	1.8	1	2.6	0	0.0	0	0.0
Arquivos de Neuro-Psiquiatria	1	1.8	1	2.6	0	0.0	0	0.0
Perception	1	1.8	1	2.6	0	0.0	0	0.0
Acta Psychologica	1	1.8	1	2.6	0	0.0	0	0.0
Journal of Experimental Psychology: Human Learning and Memory	1	1.8	1	2.6	0	0.0	0	0.0
Journal of Memory and Language	1	1.8	1	2.6	0	0.0	0	0.0
**CITATIONS GOOGLE SCHOLAR**								
Up to 50	32	58.2	20	51.3	10	71.4	2	100.0
51–100	9	16.4	6	15.4	3	21.4	0	0.0
101+	14	25.5	13	33.3	1	7.1	0	0.0
**CITATIONS SCOPUS**								
Up to 50	38	69.1	25	64.1	11	78.6	2	100.0
51–100	6	10.9	4	10.3	2	14.3	0	0.0
101+	11	20.0	10	25.6	1	7.1	0	0.0
**CITATIONS WoS**								
Up to 50	40	72.7	25	64.1	13	92.9	2	100.0
51–100	4	7.3	4	10.3	0	0.0	0	0.0
101+	11	20.0	10	25.6	1	7.1	0	0.0
**PAPER AVAILABILITY**								
Available online	54	98.2	38	97.4	14	100.0	2	100.0
Conditionally available online	1	1.8	1	2.6	0	0.0	0	0.0
**DATASET AVAILABILITY**								
Freely available	42	76.4	30	76.9	10	71.4	2	100.0
Conditionally available	4	7.3	3	7.7	1	7.1	0	0.0
Not available	9	16.4	6	15.4	3	21.4	0	0.0

**Table 2:** Generic definitions of relevant dimensions, examples of instructions and their scales.

**Dimension**	**Short definition**	**Instruction example**	**Scale**
Aesthetic appeal	The pleasantness of the image	Participants are asked to consider how visually appealing the image is in regard to its visual characteristics.	1-visually unpleasant/unappealing to 7-visually pleasant/appealing
Age-of-acquisition	The estimated age of learning a given concept/name	Participants are invited to estimate the age they thought they learned each of the concept names in its written or oral form.	age ranges from 0 to 12 years old (with different intervals)
Arousal	The activation capacity of the object	Participants have to indicate to which extent an object represents something active/intense or passive/calm.	1-very passive/calm to 7-very active/intense
Category agreement	The most appropriate category	Participants have to indicate the object category (e.g., to identify a “car” as part of the category “vehicles”). If they are unable to identify a category, they have to indicate that they don't know or they know but do not remember the name at the moment.	% or H-value (written/typed/oral form; in some cases, can be done as forced choice).
Familiarity	The frequency of the object in the participant's personal life, that reflects the likelihood of encountering the item in everyday life	Participants are asked to consider how often they encounter the item represented in the picture in their daily-life, indicating how familiar the stimulus is.	1-unfamiliar to 7-very familiar
Image Agreement	The imageability of the concept and its agreement with the picture	Participants are invited to elaborate a mental image based on a concept and, subsequently, rate if the picture presented match the previous formed mental image.	1-low agreement to 7-high agreement
Manipulability	The level of interaction required by the object	Participants are invited to rate each item/object based on the degree to which the object requires the use of a human hand to perform its function.	1-never necessary to 7-totally indispensable
Name agreement	The most common name/modal name	Participants are invited to provide in one or more words what they think is the best name for the item/object represented in the picture as fast and accurately as possible. When they are not able to provide a name, they have to indicate if they don't know or if they recognize the object but are not able at the moment to remember its name.	% or H-value (written/typed/oral form)
Picture-name agreement	The congruence between the image and the name	Participants are asked to evaluate the goodness of an image in representing the name presented.	1-very poor representation of the name to 7-excellent representation of the name
Typicality	The representativeness of the item in its own category	Participants have to evaluate if the object represented in the picture is a good example of the category presented, regardless of the occurrence of the object in their everyday life or their personal preferences.	1-very bad example of its category to 7-excellent example of its category
Valence	The pleasantness or emotional weight of the object	Participants are requested to evaluate if the item/object refers to something positive/pleasant or negative/unpleasant.	1-very negative/unpleasant to 7-very positive/pleasant
Visual complexity	The amount of visual details of an image	Participants have to evaluate to which degree the picture is easy to reproduce, in regard to the amount of visual details (e.g., lines, colors) considering the picture itself and not the actual object/concept represented.	1-very simple to 7-very complex

### Bibliometric Information and Indicators

**Figure 2:**
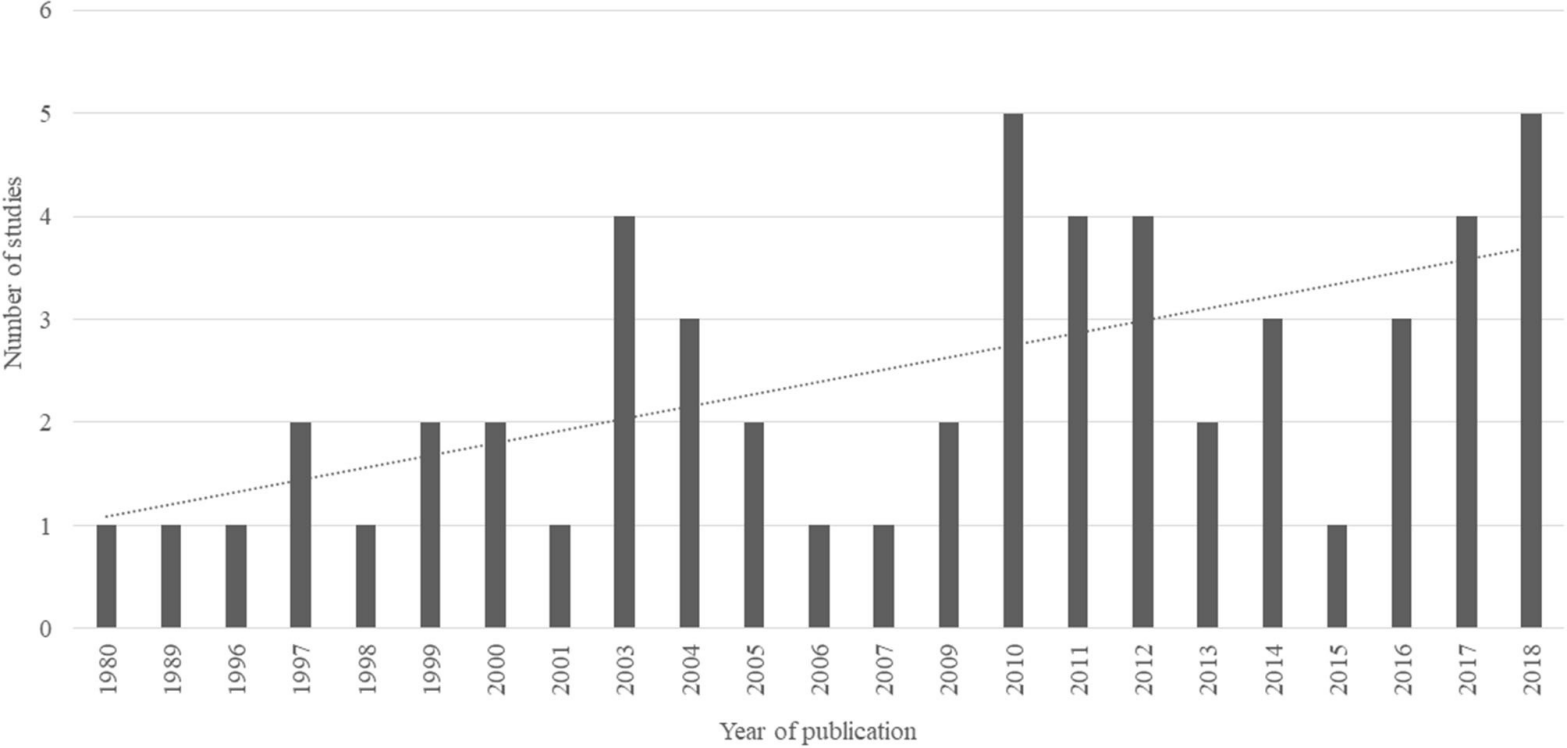
Temporal distribution of reviewed normative studies using common objects across years (%).

The evolution over the years of published normative studies using images of common objects is notable, although the number of studies is still scarce. The studies selected were published in the last 38 years (from 1980 to 2018), but mostly in the last 10 years (47 studies, 85.45%, between 2000 and 2019; see Figure 2). The main journal for publishing such type of articles was *Behavior Research Methods* (*n* = 27), which is not surprising given the scope of this publication. However, several recent normative studies with photos have been published in open access journals, such as *PLoS ONE, Frontiers in Psychology* and *Frontiers in Human Neuroscience*. To assess the impact of the journals in which the reviewed articles were published, the h-indexes were obtained using the SCImago platform (www.scimagojr.com) (see Masic, 2016). The h-index values obtained during March 2020 ranged from 22 to 268. Moreover, only three journals were ranked below h-index of 50 and one journal did not present h-index values. A google h5-index is also provided. These indicators suggest that normative studies using images of common objects have been increasingly published in the last years in relevant peer-reviewed journals (see Table 1 and Supplementary Material S3).

### General Characteristics and Standardization Practices of Images of Common Objects

The main characteristics of the revised studies were organized into four subsections: Sample characteristics; Language and cultural variations; Procedures; Stimulus characteristics. The main results are summarized in Table 1 (for detailed descriptions, please see the Supplementary Material S3).

#### Sample Characteristics

Overall, the majority of the studies (*n* = 42; 76.4%) reviewed used samples of University students (i.e., undergraduate, graduate, post-graduate levels, or both), with fewer studies recruiting participants outside the academic environment (e.g., Boukadi et al., 2016). Some studies did not provide specific information about the education level of their samples (*n* = 12; 21.8%). This review also indicated that most of the studies included young adults (with ages between 18 and 35 years old) and only 14 studies (24%) included broader age samples (e.g., larger age ranges as Brodeur et al., 2010, 2014, or age subsamples, as George and Mathuranath, 2007). Notably, there were studies in which detailed age-related information was not provided (*n* = 13; 24%). Studies using photographs were mostly conducted with undergraduate student samples, with a narrow age range (see Supplementary Material S3). While some cognitive abilities are known to decline with age and to vary with education level (Faubert, 2002; Brucki and Rocha, 2004), the current review indicates that the comparison between different education levels and different age groups in normative studies of images of common objects was not frequent, particularly for more ecological stimuli (see Table 1). None of the 55 elected studies reported education differences and 12 from the 14 studies using samples from different developmental stages, such as children, young adults, older adults, considered age variability for at least one of the dimensions (Berman et al., 1989; Cycowicz et al., 1997; Morrison et al., 1997; Ferraro et al., 1998; Pind et al., 2000; Pompéia et al., 2001; Yoon et al., 2004; Sirois et al., 2006; George and Mathuranath, 2007; Liu et al., 2011; Ghasisin et al., 2014; Saryazdi et al., 2018). In such cases, the exploration of the differences between adults vs. older participants (Ferraro et al., 1998; Yoon et al., 2004; Ghasisin et al., 2014) or adults vs. children (Berman et al., 1989; Cycowicz et al., 1997; Pompéia et al., 2001) were referred. Sirois et al., 2006, reports sociodemographic-based norms. Other studies controlled the impact of sociodemographic information, like age, schooling, and gender differences (Kremin et al., 2003; Moreno-Martínez et al., 2011; Moreno-Martínez and Montoro, 2012).

#### Language and Cultural Variations

In the 55 studies reviewed, 16 distinct languages were considered for standards and contemplated a variety of contexts (e.g., Dutch in the Netherlands and in Belgium, Duñabeitia et al., 2018, and Portuguese from European and Brazilian contexts examined in Prada et al., 2010, 2014, and Pompéia et al., 2001, respectively). Native speakers of English (*n* = 14; 25.5%) were the most recruited samples. Other languages referred across the study sample were: Indian, Greek, Persian, Icelandic, and Thai. Only, five studies (9.1%) examined more than one language/culture and three did not specify the native language of the sample. The 14 studies using photographs of common objects were mostly conducted in English, Spanish, and Portuguese (*n* = 3, each; 64.2%), and the remaining in other language communities (see Supplementary Material S3). The advances in the field are also reflected in the increased variety of languages/cultures in which recent norms have been produced (see Table 1).

While most studies presented a contrast with other normative results (i.e., between studies) for validity and reliability purposes, studies with a specific purpose of cross-cultural comparisons (i.e., collecting data in the same study for the same dataset using samples from distinct cultures) were rare. In addition to the scarce examination of cross-linguistic/cultural reported in the entire sample of studies (Kremin et al., 2003; Székely et al., 2004; Yoon et al., 2004; Torrance et al., 2017; Duñabeitia et al., 2018), from the studies using photographs only the BOSS database was evaluated across different native languages and cultures in distinct studies (i.e., English-Canadian—Brodeur et al., 2010; French—Brodeur et al., 2012, 2014; Thai—Clarke and Ludington, 2018).

#### Procedures

Overall, data collection procedures included multiple tasks with careful and systematic procedures (i.e., controlling presentation times, using well-designed stimuli, balancing the number of stimuli per participant, previously planned task order, inspecting co-occurring variables, consistency in instructions and ratings, applying consistent measures) to avoid fatigue and bias in the ratings across dimensions (see Snodgrass and Vanderwart, 1980; Rossion and Pourtois, 2004; Adlington et al., 2009; Brodeur et al., 2012, 2014; Nishimoto et al., 2012; Shao and Stiegert, 2016). In the majority of the studies all or a large number of items (up to 200) were evaluated by the same participants in a limited number of dimensions. However, in some of the studies, participants were asked to evaluate a smaller subsample of images in a wider range of dimensions (see Foroni et al., 2013 for an example).

Recent studies have also been using more controlled designs and more sophisticated experimental procedures (e.g., controlling presentation times and inter stimulus intervals), even when response times were not a variable of interest (although such concerns were already present in Sanfeliu and Fernandez, 1996; Prada and Ricot, 2010; Moreno-Martínez et al., 2011; Saryazdi et al., 2018). Recently, we have also been witnessing the emergence of alternative procedures in data collection with the use of online platforms (Survey Monkey, Qualtrics, Creative Commons, Google form, Amazon Mechanical Turk, etc.). In the present studies sample, the use of in-lab surveys and experimental procedures was predominant and only two (Székely et al., 2003; Saryazdi et al., 2018) out of the 55 studies used some online tool for collecting data. Specifically, in the study of Saryazdi et al. (2018) the goal was to compare norms produced using online and in-lab collection procedures, and they attested the similar quality of these practices. Although more studies are required to confirm it, these online resources seem promising in overcoming emerging obstacles in recruiting participants for such extensive studies.

#### Stimuli Characteristics

The selected studies included different types of common objects stimuli, using line-drawings (*n* = 39; 71%) or photographs (*n* = 14; 25.4%) or both (*n* = 2; 3.6%). From the line-drawing's studies sample, the majority (*n* = 29; 74.4%) included the Snodgrass and Vanderwart (1980) or some adaptation/extension. The remaining line-drawing studies used other stimuli created by the authors or selected from other sources (e.g., Ferraro et al., 1998; Duñabeitia et al., 2018). Among the photographs' studies sample, half used stimuli from the BOSS database (7; 50%) and the remaining used variations of common objects as stimuli embedded in contextual scenes (Shao and Stiegert, 2016), modified versions of object images (Prada and Ricot, 2010) and animals with negative valence (Prada et al., 2014). Moreover, two studies produced norms for both line-drawings and colored photographs (O'Sullivan et al., 2012; Saryazdi et al., 2018) comparing norms from Snodgrass and Vanderwart's (1980) and the BOSS (Brodeur et al., 2010, 2014) databases. These two datasets were also identified as the most used in the whole sample.

As for the number of stimuli in each database, the 55 studies reviewed ranged from 50 (Prada et al., 2014) to 930 (Brodeur et al., 2014) stimuli. This range was also observed in the 14 studies using only photographs of common objects (Supplementary Material S3). Another feature of stimuli characteristic is image quality, which is a specific and important concern in studies using more realistic images (i.e., high-quality photographs). Our analyses indicated an absence of standards for image resolution across studies (ranging from 150 × 150 to 2,000 × 2,000 pixels), with almost half (45.5%) of the articles missing this specific information. Critically, objective assessments of image quality parameters (color—RGB and luminance) have been scarcely addressed (Foroni et al., 2013; Shao and Stiegert, 2016; Forsythe et al., 2017). Likewise, the dimensions of color diagnosticity (Rossion and Pourtois, 2004; Adlington et al., 2009) and goodness of depiction (Székely et al., 2003) are almost absent. However, a few recent studies have been implementing specific procedures to produce high-quality photos of common objects controlled for their surface parameters (Brodeur et al., 2012, 2014; Saryazdi et al., 2018). Other recent studies (Forsythe et al., 2017; Torrance et al., 2017) also used automated measures of visual complexity. The use of refined measures for surface parameters of the images constitutes an improvement in standardization practices since the classic Snodgrass and Vanderwart norms 1980 and requires sophisticated technological resources that are currently available (i.e., scripts and image processing programs).

Finally, the majority of the studies distributed the stimuli into categories (e.g., animals, vegetables, and tools; verbs and nouns). The number of categories varied across studies ranging from one broad concept (i.e., concrete names in Paolieri and Marful, 2018) to 32 distinct categories (i.e., Brodeur et al., 2014) that included concepts from living and non-living domains. Overall, the studies used a low to moderate number of categories, with 31 studies referring between 6 and 15 categories (56.4%) and 8 studies including <5 categories (14.5%). However, studies reporting <5 categories only made generic reference to categories and/or domains. For example, Berman et al. (1989) used one general category to group basic-level concepts (e.g., dolphin, chair) and Sirois et al. (2006) used the living domain as a unique animate macro category in contrast to man-made, body parts and professions as inanimate categories. Only seven studies reported more than 15 categories (see Table 1), particularly when norming photographs. Some authors also considered the item distribution into the categories by domains. Moreno-Martínez and Montoro (2012), for example, presented 10 categories from the living domain (e.g., birds, insects) and 12 categories from the non-living domain (e.g., weapons, tools). Also, the norms by Prada et al. (2010) included four categories containing items from the living and six from the non-living domain.

The semantic organization effect of categories and domains (living vs. non-living) across dimensions was not consistently examined, particularly in those using real-world photographs (but see Magnié et al., 2003; Laiacona et al., 2016). Moreover, the few normative studies that systematically explored those effects presented interesting results showing the influence of distinct semantic content across specific dimensions (Magnié et al., 2003; Rossion and Pourtois, 2004; Adlington et al., 2009; Brodeur et al., 2012; Foroni et al., 2013; Laiacona et al., 2016; Clarke and Ludington, 2018). Of special relevance, Adlington et al. (2009) provided evidence for the effect of semantic organization on naming performance (with better naming for categories from non-living things) as well as the modulation of this effect by gender (women were better at naming living things while men were more accurate in naming non-living things).

### Dimensions Reported in the Standardization of Images of Common Objects

The final sample of 55 studies was then examined regarding the dimensions consistently reported from the semantic (i.e., Name-agreement; Category-agreement; Familiarity; Typicality), perceptual (i.e., Visual complexity Picture-name agreement), and affective (i.e., Aesthetic appeal; Arousal; Valence) domains (see Table 2 for generic definitions, examples of instructions and scales for each relevant dimension).

The inspection of the instructions and measures of the most referred dimensions revealed some consistency across studies, mostly for name agreement and visual complexity tasks. However, the instructions for familiarity and image agreement dimensions, differed in their focus (e.g., on picture, object or concept) or in some cases presented inconsistencies between the instruction focus (e.g., concept-based) and scale (e.g., object-based) (see Bonin et al., 2003; Janssen et al., 2011). The instructions also varied reflecting the developments on the definition of the dimensions. For instance, studies evaluating familiarity based on encounter/frequency and also examining image agreement based on object agreement and viewpoint were documented (see Brodeur et al., 2010 for an example). The necessity to disentangle dimensions is also referred in some normative studies, when comparing different definitions of the same dimension (e.g., Adlington et al., 2009 measurement of familiarity based on picture vs. based on the concept) or contrasting potentially confounding dimensions (e.g., Snodgrass and Vanderwart, 1980 examination of Image agreement and Picture-name agreement). These issues have been recently addressed in attempts to provide more specific definitions, such as the clearer definition of familiarity presented in Saryazdi et al., 2018, or the requirement for a specific name (Moreno-Martínez et al., 2011) or for the most correct spelling of first language labels (Torrance et al., 2017) in name agreement. The comparative table of instructions from the most reported dimensions and their scales across studies can be found in Supplementary Material S4, Table 1.

Among the semantic dimensions addressed in the 55 studies retained, norms for Name-agreement were quite frequent across studies (87.27%), in both articles norming photographs and line-drawings. Only a few exceptions did not examine this dimension (e.g., Ferraro et al., 1998; Forsythe et al., 2017). The main measures considered for Name-agreement were: the modal name (the name more frequently reported and its percentage of agreement) and the h-index (a statistical score that takes into account the influence of the number of correct names given for each item and their frequency). Notably, a recent study from Torrance et al. (2017) established norms and procedures for a variety of innovative dimensions, such as naming abilities by adding typed name; spelling agreement index (that follows the same rational of the h-index used in naming but for spelling variations); modal spelling; timing of written naming; and length of modal name. Additionally, in some of the studies the naming task was previously applied to a different sample as a pre-validation study (e.g., Brodeur et al., 2010; Clarke and Ludington, 2018). Although less usual, an *a priori* judgment procedure (see Khwaileh et al., 2018) might constitute a good alternative for previously defining the name and the cultural appropriateness. This procedure usually involves different judges (linguistic experts or culturally-based elected) invited to evaluate the items independently (e.g., name, category, quality of the image, etc.).

Norms for Familiarity were reported in 83.64% of the 55 studies (e.g., Cuetos et al., 1999; Bonin et al., 2003; Zhou and Chen, 2017). Familiarity was always reported in studies using photographs (see Supplementary Material S3). Age-of-acquisition was reported in almost half (49.09%) of the studies. In contrast, only 9.09% (e.g., Dell'Acqua et al., 2000) of the studies considered Typicality ratings from which three where from norms for photographs (5% of the 14 studies). In most normative studies (92%), categories were previously defined by the researchers using mainly superordinate and basic-level categories. Category-agreement was not explored in line-drawings and only four studies with photographs evaluated this dimension.

Regarding the perceptual dimension, Visual Complexity (*n* = 33 out of 55, 60%; *n* = 10 out of 14 with photos, 71%) and Image Agreement (*n* = 19 out of 55, 34.55%; *n* = 4 out of 14; 28%) were the most reported dimensions. Moreover, Imageability (*n* = 8 out of 55; 14.55%; *n* = 1 out of 14; 7%) and Picture-name agreement (*n* = 4 out of 55; 7.27%; *n* = 0 out of 14; 0%) were also examined. Additionally, a few studies (*n* = 7; 13%) addressed Manipulability, particularly for objects and tools (i.e., Magnié et al., 2003; Brodeur et al., 2010, 2012, 2014; Moreno-Martínez et al., 2011; Moreno-Martínez and Montoro, 2012; Laiacona et al., 2016). While providing norms for Manipulability using photos of common objects, Moreno-Martínez et al. (2011) showed the significant influence of this dimension in other variables, such as naming, h-index, familiarity and visual complexity. Several other perceptual-related dimensions were also reported but were scattered across studies (e.g., color diagnosticity, vividness, viewpoint agreement).

Affective dimensions were scarcely reported across studies, with Arousal (*n* = 1, 1.82%) and Valence (*n* = 4 out of 55; 7%) being examined but only in real-word photographs of common objects (Prada and Ricot, 2010; Prada et al., 2010, 2014; Foroni et al., 2013). However, from those studies only Foroni et al., 2013, was retrieved from the automatic search. Other Affective/Emotional dimensions (i.e., disgust, fear, dangerous) and Beauty were only documented in one study each (Magnié et al., 2003; Prada et al., 2014, respectively). Norms for Aesthetic Appeal were not reported in the elected studies, even though this dimension is known to significatively influence the processing of visual items (Reppa and McDougall, 2015).

**Figure 3:**
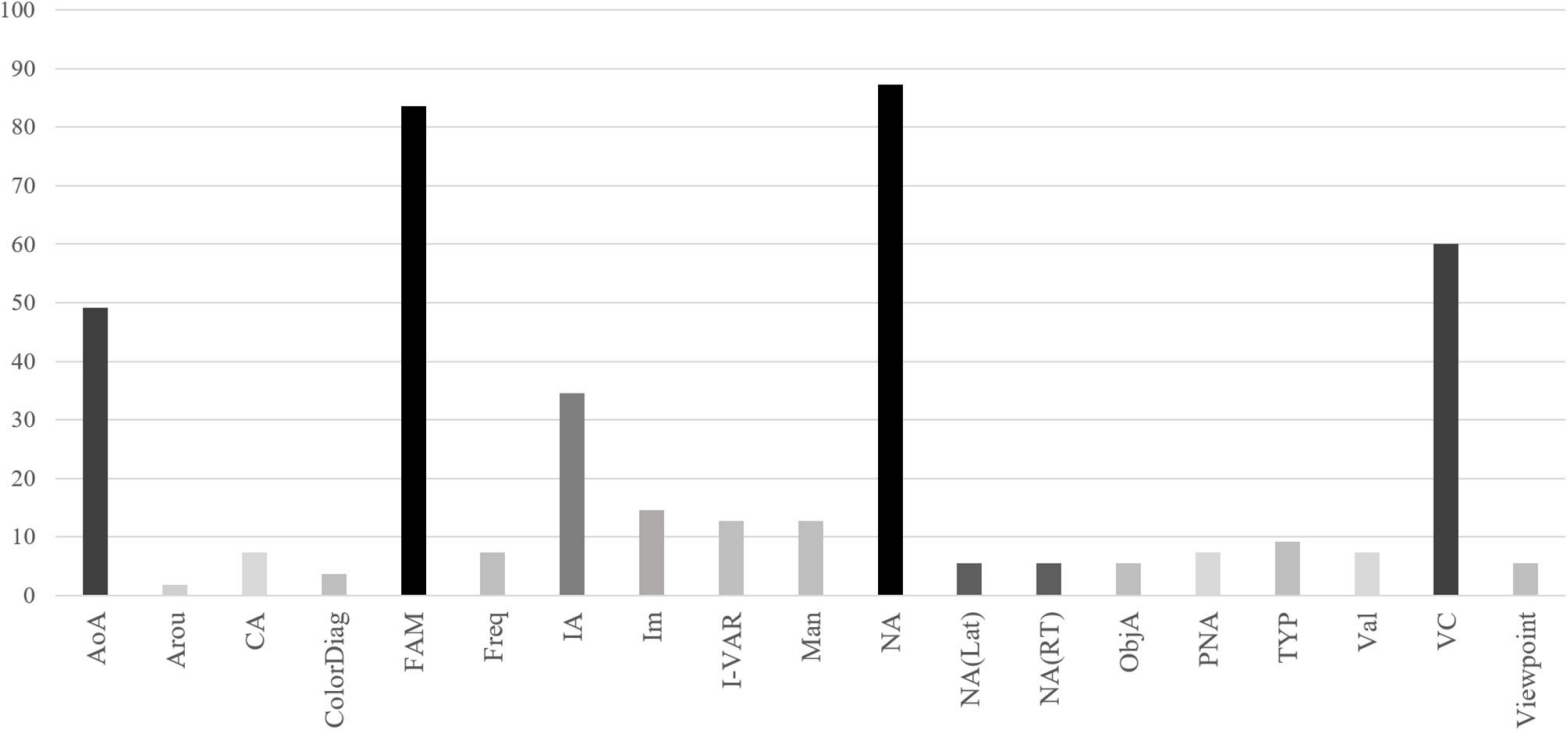
Representativeness of each dimension across studies (%).

Finally, there were some other dimensions sporadically addressed across studies, as action content, ambiguity, image variability, body-object interaction, vividness, index recollection, verb generation, word length, as subdimensions of familiarity, such as frequency of the concept and likelihood of the object in daily life (see Barry et al., 1997; Kremin et al., 2000; Saryazdi et al., 2018) but they remain rather unexplored in images of common objects norms. The distribution of the dimensions across studies is presented in Figure 3.

### Assessment of Images of Common Objects

#### Qualitative Appraisal of Norms

A qualitative inspection of the ratings across studies indicated that images of common objects are rated as moderately to highly familiar (Rossion and Pourtois, 2004, but see also Pompéia et al., 2001; Brodeur et al., 2012, 2014; Moreno-Martínez and Montoro, 2012; Raman et al., 2014; Shao and Stiegert, 2016), and low to moderate in complexity (e.g., George and Mathuranath, 2007; Adlington et al., 2009; Dimitropoulou et al., 2009; Brodeur et al., 2014; Shao and Stiegert, 2016). A study contrasting both types of items showed that photos obtained higher name agreement and picture-name agreement scores as well as lower familiarity, visual complexity and less variability in naming (h-value) than line-drawings stimuli (see Saryazdi et al., 2018). The majority of the studies reported a reasonable agreement (higher than 65%) regarding their modal name (e.g., Cuetos et al., 1999; Nishimoto et al., 2012; Paolieri and Marful, 2018). However, the h-value of naming presented a high range across studies. Category agreement was higher than 68% across studies, although few studies reported norms on this dimension (Brodeur et al., 2012, 2014), and typicality was rated as moderate to high (Moreno-Martínez et al., 2011; Moreno-Martínez and Montoro, 2012). Age-of-acquisition was measured in different ways across studies (e.g., some studies used 2 or 3 age bands and others simply asked to type that age). The ratings of valence and arousal were not enough to capture possible trends in the reports across studies (Prada et al., 2010). Moreover, arousal and valence showed to be sensitive to category variations (i.e., tools, animals, vegetables) and also to vary depending on typicality and familiarity ratings (Foroni et al., 2013).

Overall, and despite the relevance of mapping the distribution of evaluative scores across dimensions, these trends should be interpreted with caution and consider the specific characteristics of the database normed. See Supplementary Material S4, Table 2, for obtaining the distribution of scores by study.

#### Correlations

All the reviewed studies that report correlations are referring to associations of some semantic (predominantly, name-agreement and familiarity) and perceptive dimensions (mostly, visual complexity), but affective variables were rarely examined. Additionally, none of the reviewed studies simultaneously explored the relations between dimensions of these three domains. The most frequent combinations were the perceptive and semantic domains. The semantic and affective or perceptive and affective combinations were also found, albeit scarce. Interestingly, the simultaneous examination of affective and other domains was only present in photographs of common objects norms. The results of the association between dimensions in the reviewed studies and the comparisons between stimuli type (line-drawings vs. photographs) are presented in Table 3.^2^

**Table 3:** Significant correlations between dimensions in normative studies of line-drawings and photographs.

	**AoA**	**CA**	**Fam**	**IA**	**I-var**	**NA%**	**NA(H)**	**PNA**	**Typ**	**VC**
AoA		NR	−0.91^**^^p^ −0.37^**^^q^	−0.13^*^^s^	NR	−0.25^**^^q^ −0.82^***^^u^	0.17^*^^t^ 0.75^**^^p^	NR	−0.72^**^^r^ −0.91^**^^p^	−0.26^**^^p^ 0.34^**^^x^
CA	NR		0.30^*^^r^ 0.22^**^^q^	0.14^**^^q^	NR	NR	−0.93^**^^p^	NR	NR	NR
Fam	−0.38^**^^a^ −0.64^***^^b^	NR		−0.19^**^^s^ 0.46^**^^t^	NR	0.35^**^^q^ 0.89^***^^u^	−0.29^**^^v^ −0.71^***^^u^	NR	0.75^**^^x^ 0.92^**^^p^	−0.21^**^^q^ −0.57^**^^x^
IA	−0.30^**^^c^ −0.14^**^^d^	NR	−0.15^**^^e^ 0.44^*^^k^		NR	0.46^**^^q^ 0.49^**^^t^	−0.17^**^^s^ −0.43^**^^q^	NR	NR	−0.19^**^^r^
I-var	−0.24^**^^d^ −0.64^**^^e^	0.19^**^^e^ 0.62^*^^j^		−0.17^**^^h^ −0.29^**^^k^		NR	NR	NR	NR	NR
NA%	−0.16^*^^f^ −0.52^**^^d^	NR	−0.57^*^^l^ 0.52^**^^m^	0.20^*^^l^ 0.49^*^^c, n^	0.20^**^^a^ 0.32^**^ *^*d*^*		−0.83^***^^u^ −0.96^*^^r^	NR	0.52^**^^x^ 0.73^**^^p^	−0.11^*^^r^ −0.26^***^^u^
NA(H)	−0.18^***^^e^ −0.57^**^^g^	NR	−0.15^*^^n^ −0.39^**^ *^*m*^*	−0.24^**^^a^ −0.55^***^^n^	−0.14^*^^j^ −0.28^**^^k^	−0.74^**^^k^ −0.96^***^^n^		NR	−0.51^**^^z^ −0.66^**^^p^	NR
PNA	−0.08^*^^f^ −0.14^**^^b^	NR	−0.21^**^^k^	0.72^**^^k^	−0.35^**^^k^	0.12^*^^f^	−0.25^***^^b^		NR	NR
Typ	NR	NR	NR	NR	NR	NR	NR	NR		−0.27^**^^x^ −0.26^**^^p^
VC	−0.25^*^^h^ 0.53^***^^i^	NR	−0.12^*^^o^ −0.48^**^^c^	−0.14^*^^k^ −0.59^**^^n^	−0.17^*^^h^ −0.24^*^ *^*j*^*	−0.15^**^^h^ −0.49^***^^n^	−015^**^^c^ 0.54^***^^n^	−0.22^***^^b^	NR	

The correlations are significant at

* p < 0.05;

** p < 0.01;

*** p < 0.001. The signal (–) is reported for negative direction of the correlations.

The photographs (10) results are presented above the diagonal and line-drawings (28) results are presented below the diagonal of the table. Only the maximum and minimum correlational results for each correlation across studies are presented. AoA, Age-of-acquisition; CA, Category agreement; Fam, Familiarity; IA, Image Agreement; I-var, Image variability; NA%, Name agreement percentage; NA(H), Name agreement H-value; PNA, Picture name agreement; Typ, Typicality; VC, Visual complexity. NR, not reported across studies. Affective dimensions (i.e., arousal, aesthetic appeal, and valence) were not reported in this table, once there were no studies reporting such dimensions in line-drawings studies samples.

Line-drawing references:

a Bonin et al., 2003;

b Johnston et al., 2010;

c Raman et al., 2014;

d Alario and Ferrand, 1999;

e Liu et al., 2011;

f Morrison et al., 1997;

g Nishimoto et al., 2005;

h Manoiloff et al., 2010;

i Sirois et al., 2006;

j Nishimoto et al., 2012;

k Sanfeliu and Fernandez, 1996;

l George and Mathuranath, 2007;

m Boukadi et al., 2016;

n Tsaparina et al., 2011;

o Khwaileh et al., 2018.

Photographs references:

p Moreno-Martínez et al., 2011;

q Clarke and Ludington, 2018;

r Brodeur et al., 2010;

s Shao and Stiegert, 2016;

t Paolieri and Marful, 2018;

u Adlington et al., 2009;

v Zhou and Chen, 2017;

x *Moreno-Martínez and Montoro, 2012*.

Overall, the correlations scores indicate consistency in the direction of the correlations across studies, with a few exceptions [photos: NA(H)-AoA, NA%-FAM, VC-AoA; line-drawings: VC-AoA, IA-FAM]. Correlations between semantic dimensions were overrepresented. Name agreement measures were negatively correlated for both type of items. The correlation between name agreement (%) and familiarity was positive and from moderate to strong, independently of the stimuli type. Moreover, visual complexity was negatively correlated with familiarity (see Raman et al., 2014; Clarke and Ludington, 2018) and name agreement (Tsaparina et al., 2011) while picture-name agreement was positively related to image agreement (Sanfeliu and Fernandez, 1996) (see Table 3). In line with previous findings using word stimuli (see Santi et al., 2015), typicality showed a positive correlation with familiarity and with name agreement (%) for photographs. In perceptive dimensions, visual complexity was negatively correlated with typicality, familiarity and image agreement, although its association with name agreement (H) and with category agreement remained absent (Table 3). Picture-name agreement was also positively associated with name-agreement. Correlations between affective dimensions are not reported, however it is noted that valence and arousal have been positively correlated in the literature for specific categories of common objects (Foroni et al., 2013).

The examination of reliability in cross-cultural comparisons was made by extracting cross-studies correlational data from the studies sample that reported the comparison of each relevant dimension with other studies using direct analysis (i.e., with the very same images). Overall, the most reliable dimensions across studies comparisons were age-of-acquisition and image agreement. In such dimensions, the correlations found (between moderate to strong) represented a comparison between very distinct cultural and linguistic contexts (i.e., Russian and American). The high variability on Naming agreement scores indicates their sensibility to changes in cultural/linguistic variations (e.g., Tunisian Arabic vs. Spanish) and present strong correlations in similar linguistic backgrounds (i.e., American vs. British English). The results extracted from the articles reporting correlations between the original (reference) and previous studies using the same database are presented in Table 4 (extracted results by article can be found in Supplementary Table 3).

**Table 4:** Descriptive information of cross-country comparisons of the dimensions across studies.

**Dimension**	**Correlation range**	**Qualitative range**	**Direction**	***N***	**Strong correlation (Freq.)**	**Strong correlation (%)**
NA (h)	0.15–0.69	Weak to moderate	+	24	0	0%
NA (%)	0.15–0.74	Weak to strong	+	24	2	8%
FAM	0.27–0.99	Weak to strong	+	22	17	77%
AoA	0.56–0.95	Moderate to strong	+	16	10	63%
IA	0.42–0.83	Moderate to strong	+	12	4	33%
VC	0.38–0.92	Moderate to strong	+	20	15	75%

Only significant results (p < 0.05) were considered for this analysis; (–) are and (+) indicate the direction of the correlations.

*NA, Name-agreement; AoA, age-of-acquisition; IA, Image agreement; FAM, Familiarity; VC, Visual Complexity were considered based on their high occurrence across studies*.

### Availability and Application Potential of the Normative Databases

In order to evaluate the potential use of the databases, we collected information about their availability and their application.

From the 55 articles retained, the majority were available online (98%) and presented free access to the database (*n* = 43, 78%) and only eight (14%) did not refer how to access the database or presented an unavailable link (e.g., Dell'Acqua et al., 2000; Kremin et al., 2003; Bayram et al., 2017). Four studies (7.3%) allowed the conditional access to the database (Dimitropoulou et al., 2009; Janssen et al., 2011; Moreno-Martínez et al., 2011; Raman et al., 2014) controlled by the Editors/Journals website or by the first authors (see Table 1).

The number of citations of the articles in the Web of Science (range: 0–3,947), Scopus (range: 0–4,023), and Google Scholar (range: 0–5,783) is relatively high. However, a closer inspection to these numbers revealed that there are very few articles with more than 100 citations (*n* = 14 in Google Scholar, 25.5%, and *n* = 11 in Scopus and WoS, 20%). The most cited articles from the overall sample refer to normalization of line-drawing images (Snodgrass and Vanderwart, 1980; Morrison et al., 1997; Rossion and Pourtois, 2004). As expected, the recency of a publication is likely to reduce its citation scores (see Table 1). Therefore, it is not surprising that the articles with fewer citations were those reporting norms for photographs of common objects that also constituted the most recent publications. However, even considering average citation per year indicators, the studies norming line-drawings are still the most cited ones (see Supplementary Material S3). These findings are somehow surprising giving the increasing and extensive interest in ecological stimuli.

## Discussion

Several normative studies in the psychological field have already established criteria to examine how specific variables are stated/evaluated in a sample of interest (Cicchetti, 1994). The assessment of any construct or variable, particularly in experimental studies, requires that the measuring tools designed for such assessment are efficient (i.e., validity) in producing reliable results. Therefore, standardization of procedures, materials and scores are essential to avoid undesirable interferences in psychological assessment (see Fischer and Milfont, 2010). To this end, several standards for building and normalizing measurement tools as well as rich statistical resources have been made available (Cicchetti, 1994; Fischer and Milfont, 2010). Kyriazos and Stalikas (2018) presented relevant steps on scale development to guarantee their quality, such as the theoretical framing of the variables of interest, adequate measures of assessment (i.e., response type scale and psychometric properties) and also item quality (i.e., development and selection of good exemplars). However, specific guidelines for normative procedures of stimuli production and their selection for research/interventional purposes have been scarcely discussed. Indeed, this type of standardization reveals itself as a potential research field that remains rather unexplored.

In response to this gap, the present review evaluated the current status of normative studies using images of common objects with adults in order to systematically map and characterize the main features and practices in the field. The information extracted from the retrieved studies, was coded and summarized to answer the proposed research questions namely: (a) bibliometric information and indicators (journals, journals h-index, temporal distribution); (b) general characteristics and standardization practices of images of common objects (sample characteristics, language and cultural variations, procedures, stimulus characteristics); (c) dimensions reported in the standardization of images of common objects (main dimensions reported, scales and task instructions, and their consistency across studies); (d) assessment of images of common objects (mean ratings and correlational results, reliability of datasets); (e) accessibility and application potential of the normative databases (availability and citation impact).

Overall, the results indicated 55 published normative studies using images of common objects. The bibliometric indicators examined revealed that normative studies of images of common objects have been increasing in the last 10 years and published in quality peer-review journals. These indicators document the recent efforts that have been made in the field to provide stimuli and to produce valid norms that support adequate manipulations and enhance quality and replicability in experimental research (Wilcox and Claus, 2017). However, their use should consider systematic and contextualized knowledge about the databases, their dimensions and normalization procedures.

The general characteristics and standardization practices of images of common objects, indicated that the reviewed studies were conducted with healthy young and highly-educated adults. The sampling procedure is probably one of the most important steps during normative studies. Once the samples constitute a reference to produce norms, their characteristics must be representative of the population (e.g., age, gender, language, nationality, QI, education level, etc.), and the sample size constitutes an important criterion for statistical purposes (Cohen, 1988; Mitrushina et al., 2005). Although a restricted sample may preclude generalized conclusions, the widespread use of academic samples, such as those reported in most of the reviewed articles may favor the comparison across normative studies (Pompéia et al., 2001; Garrido and Prada, 2017) and be suitable for the large number of experimental studies that are often conducted with University students.

Another feature of the samples in the reviewed studies is their limited age distribution and consequent scarcity of aging and development effect analysis. In conducting norms for adult populations, it is relevant to fully consider their developmental process once with increasing age some abilities (i.e., perceptual and memory) are known to decrease (for a review see Faubert, 2002) while others may increase with life experienced knowledge (as vocabulary, see Verhaeghen, 2003 for a meta-analytic summary of findings in such topic) impacting the way norms are rated. The production of norms for images of common objects with healthy elderly participants might be important for studies using these stimuli for contrasting such population to others of the same age range but with clinical condition (see Semenza et al., 2003; Laws et al., 2007). Specifically, having standards for healthy samples of older participants may improve the quality of assessments in defining diagnostic markers and also designing interventional strategies in the clinical context. It is also worthy to note that the comparison between norms obtained with adults and norms obtained with samples from earlier stages of the life course is also scarcely presented in the reviewed studies (Berman et al., 1989; Morrison et al., 1997; Pompéia et al., 2001). The norms produced for children are crucial for understanding how development affects several dimensions and to confirm the consistency of some procedures (e.g., the adequacy of Age-of-Acquisition measures used with adults) (see Morrison et al., 1997; Pompéia et al., 2001 for an example) and, thus serve as a baseline for further research. Additionally, the production of norms with children is of great use in psycholinguistic and neurodevelopmental research, in which standardized images of common objects stimuli are frequently used. Nevertheless, there are normative studies across the life course which were not captured in our review simply because our search was restricted to young adults.

The language and cultural variations of the samples covered different linguistic and cultural environments, although they were predominantly constituted by speakers of English or other European languages. The prevalence of a linguistic/cultural background together with the scarcity of direct cross-cultural comparison reported reflects the need of enhancing country-based norms production. Cultural variations (i.e., as food habits, tools and technological resources, social rules, beliefs, religion, and especially, language) are known to influence the processing of meaningful stimuli, such as common objects (e.g., George and Mathuranath, 2007; Brodeur et al., 2012; Duñabeitia et al., 2018). For example, Duñabeitia et al. (2018) revealed country-based differences across correlated dimensions (e.g., h-index of naming and Visual complexity) although similarities between linguistic (i.e., English and German or Spanish and English) and also culturally-based comparisons (i.e., Dutch speakers from the Netherlands and from Belgium) were observed in mean ratings of common objects. Moreover, a cross-linguistic comparison between citizens speaking different languages but living in the same context (i.e., French and English speakers living in Canada) indicated a culturally-based convergence across mean ratings for a variety of dimensions (Brodeur et al., 2012). Consequently, the examination of the same dataset across languages and cultures may indicate specificities about their contextual variations as well as their commonalities.

The use of controlled designs and careful recruitment procedures observed (see Zhou and Chen, 2017; Saryazdi et al., 2018) reflected a continuous effort to extend and improve previously established norms production. To overcome time-consuming and resource demanding procedures (recruitment, materials, lab set preparation and availability, etc.), online studies of norms seem to constitute a valuable alternative without compromising the quality of the norms produced (i.e., King et al., 2014; Saryazdi et al., 2018). However, data collection practices are not yet fully taking advantage of those recent technologies.

The findings regarding stimuli characteristics indicated that line-drawings stand out as the most prevalent validated type of stimuli, although an increasing number of studies validating photographs of common objects has been recently observed. Real-world photographs are more realistic representations of the world (Moreno-Martínez and Montoro, 2012). They entail richer expressions of a set of object parameters that impact image processing, such as color, shade/luminance, angle, resolution, and form, which together with context regularities and the semantic content inherent to images (see Brady et al., 2008, 2009; Konkle et al., 2010) comprise more complex representations of the reality. Nevertheless, their detailed representations may limit the possibility of producing prototypes which might generate more ambiguity and, consequently, more difficulty in recognizing the objects (Brodeur et al., 2010). Due to their complexity, the examination of multiple parameters of perceptive characteristics and their relations with other dimensions are desirable in normative studies using such type of stimuli.

Overall, the databases identified, showed a moderate number of items and categories, a pattern that has been changing in the last 5 years where a higher number of stimuli and categories have been observed. Regardless of their importance in object processing (Warrington and Shallice, 1984; Chao et al., 1999), the effects of semantic categories and domains across dimensions were hardly reported. While most of the reviewed studies distributed items across categories, the need to include a wider range of categories became evident considering the limited number of studies reporting more than 15 categories as well as a higher number of items within categories. Moreover, the clustering of the images into domains was even more infrequent. The semantic content inherent to common objects and their specific categories are known to influence their processing (Warrington and Shallice, 1984; Martin et al., 1996; Semenza, 2009) with distinct neural structures recruited for the different categories to be processed (for details, see Moss and Tyler, 1997). Complementary, the categorical organization also exerts influence in affective dimensions, such as arousal and valence (Foroni et al., 2013) as well as in other semantic and perceptive dimensions (Brodeur et al., 2014) that varied according to the categories. The BOSS database normative studies (Brodeur et al., 2010, 2012, 2014) constitute an example of good practice, being the largest and more diversified dataset using photographs of common objects from categories from both living and non-living domains.

The dimensions reported in the standardization of images of common objects indicated that a variety of dimension were examined, mainly from semantic and perceptive domains. Overall, most normative studies report only a few dimensions with the affective dimension being the least explored. There is no systematic reporting of perceptive, semantic and affective dimensions within the same study. Interestingly, the simultaneous examination of dimensions from more than one of the semantic, affective and perceptive domains was more frequent in photographs than in line-drawings norms of common objects. According to Prada et al. (2016), the examination of various dimensions in the same image database is crucial to dissociate dimensions and avoid possible confounding effects and allows the selection of stimuli across dimensions as a function of the research interests. The unsystematic reporting of dimensions across studies, however, may limit the comparisons between studies (since studies may not present the same dimensions) and reduce their potential of application in prospective studies (e.g., when researchers need to control for specific dimensions that are not addressed).

Despite the diversity of dimensions reported across studies, some consistency was observed regarding the prevalence of naming-agreement followed by familiarity and visual complexity. Although not previously considered in the search procedures, age-of-acquisition was one of the most reported dimensions. These studies indicate the relevance of this dimension in object recognition, object naming, and semantic processing for adult samples (see Morrison et al., 1997; Johnston and Barry, 2005, 2006). Furthermore, some relevant dimensions that impact this type of items, such as Typicality, Category agreement, Aesthetic appeal, and Manipulability, were somehow neglected. The recent interest in the effects of manipulability was largely motivated by discoveries on the human mirror neuron system and related action imitation processes (Iacoboni, 1999) and since then have increasingly been examined (Kalénine and Bonthoux, 2008; Campanella and Shallice, 2010; Pobric et al., 2010). This effect was documented in studies showing that children show different perceptual and conceptual processing for manipulable and non-manipulable objects (Kalénine and Bonthoux, 2008). In fact, manipulability has been recognized as a dimension of the semantic system (see Campanella and Shallice, 2010). Accordingly, a recent multimodal approach suggested that semantic processing is influenced by a combination of modality-specific information comprising sensory, verbal and motor experiences, including the manipulability of objects, that are integrated at the anterior temporal lobe (Pobric et al., 2010). It seems that this dimension might be quite important for exploring common objects, and presents a considerable influence in item processing, as indicated in our study sample. Other dimensions, such as Aesthetic Appeal were not explored in the elected studies. This finding is at odds with evidence showing that this dimension impacts significantly the processing of visual items (Garrido et al., 2016; Prada et al., 2016) and interacts with other dimensions, such as familiarity (Prada et al., 2016) and affects visual inspection (McDougall and Reppa, 2008). Therefore, it requires further examination in norms for images of common objects.

Other dimensions often referred in the reviewed papers were naming latency, frequency, vividness, spelling agreement, beauty, and word length. Although important, some of these dimensions may not be critical in normative studies of images of common objects. Specifically, some of these dimensions seem to be particularly relevant for word processing (frequency, word length) and others may present conceptual similarities with well-reported dimensions (i.e., image agreement is similar to picture-name agreement).^3^

The inclusion of innovative dimensions, such as measures of color, manipulability, and ambiguity in image normative studies has been increasing in recent years inspired by developments in related fields. Therefore, it is important to contextualize the emergence of such dimensions in the broader scientific context. Particularly, visual cognition and picture processing research fields have been examining surface features and cognitive processing involved in object perception. For instance, the influence of surface features, such as color, amount of details or size, in the way visual items are perceived and retrieved (Biederman and Ju, 1988; Brady et al., 2009; Konkle et al., 2010) may have motivated the inclusion of color diagnosticity, objective RGB parameters or even ambiguity in normative studies. The semantic attributes in picture norms may also derive from the approach of meaningful-based top-down processes and contextual expectancies/regularities (VanRullen and Thorpe, 2001; Bar, 2003). These developments may have influenced the emergence of semantic related dimensions (e.g., based on the meaning of the items or modality of categories) associated to visual representations, such as picture-name agreement, category agreement, image agreement, manipulability, and concreteness. Others were well-explored in the word processing field but remained less explored in picture processing, namely typicality and word length. Furthermore, there were studies also reporting recent improvements in the definition of the dimensions included and related concepts. An example can be found in the familiarity dimensions that have been defined as the likelihood of being in contact or encounter an item in daily-life (see George and Mathuranath, 2007 for examples; Foroni et al., 2013). Likewise, recent findings on the difference between exposure and familiarity and the influence of exposure on perceptive processing have motivated the emergence of norms for encountered ratings (see Forsythe et al., 2017). Finally, affective dimensions have only been recently recognized as influencing the processing of non-emotional images.

The assessment of images of common objects across normative studies reviewed indicated that common objects are usually rated as moderately to highly familiar and typical and as low to moderate in complexity, with a reasonable agreement (higher than 65%) regarding their modal name and category, which vary across studies. Some of the correlations between dimensions were strong, suggesting the need for examining some potential confounds, namely between typicality and other semantic dimensions (familiarity and modal name). Moreover, unexplored dimensions, such as aesthetic appeal and arousal/valence, should be examined for their impact on the processing of images of common objects. The correlation scores provided indicators of consistency in the direction of the correlations across studies, with a few exceptions [photos: NA(H)-AoA, NA%-FAM, VC-AoA; line-drawings: VC-AoA, IA-FAM]. These exceptions seem to reflect the selective influence of context on meaningfulness-based dimensions in each stimuli type. Of special interest and despite their consistency in direction, it was observed that the range of the strength of the reported correlations presents some variation across studies, which is expected once the geographical context and language vary across studies. The results suggest that norms are sensible to both cultural and linguistic variations and their use should be restricted to the populations in which they were produced and also that they might depend on methodological differences (i.e., number of items, task instructions, data collection environment, etc.). However, the present study only examined the ratings across studies in a qualitative manner which limits the possibility of drawing substantial conclusions and generalizations on this issue.

The availability and application potential of the normative databases showed that most studies, their materials and data, have been made increasingly accessible in the last years, which favors reproducibility and application potential in the upcoming years. However, this accessibility could be further boosted, namely by increasing open access practices that facilitate the access to the stimuli, allowing replication studies across different cultural contexts and languages. Open practices are likely to stimulate the enhancement/extension of visual normative databases and the examination of new dimensions. The past use of these norms indicates a tendency to use well-established databases (Snodgrass and Vanderwart, 1980; Brodeur et al., 2010) while overlooking other more recent and less widespread ones. Furthermore, the application potential of the normative studies is acknowledged by clinical and experimental studies exploring, for example, perceptual and linguistic variables (see Funnell and Sheridan, 1992) as well as in computational and neural approaches (see Stewart et al., 2014). These databases are, therefore, extensively applied in distinct fields.

Finally, this review was also motivated by the interest in identifying potentially relevant normative datasets of images of common objects that might constitute useful resources for researchers. Choosing the adequate database depends on the goals of the research, availability of images and norms, the reported dimensions, and the context in which the norms were produced. Databases that provide norms for several dimensions and a diversity of cross-cultural examinations (e.g., Snodgrass and Vanderwart, 1980; BOSS database; Multipic), present higher application potential. The reliability of the norms by dimension constitutes one of the parameters for choosing a suitable dataset (Cicchetti, 1994; Fischer and Milfont, 2010). Furthermore, datasets of specific subcategories of stimuli that address very specific dimensions, although not focused on this review, constitute a valuable effort in guaranteeing the maximum control while selecting stimuli (i.e., FRIDa database). Based on our review, we encourage the production and use of available databases with a high number of categories and items, rated in several dimensions in different linguistic and cultural contexts.

The present review provides useful guidance for the production of norms, as well as for selecting datasets and items for experimental/interventional contexts. The selection of such stimuli for research purposes, should consider theoretical assumptions, multidimensional inspections and simultaneous control of variables outside the research focus as well as their suitability for the research question, population, and modulations of interest (see Constantinescu et al., 2016 for an example of systematic selection of stimuli within a dataset). In advancing the field, further normative studies should contemplate a diversified group of dimensions, combining dimensions and their domains (i.e., affective, semantic, and perceptive) as well as exploring them within the same image corpus and, subsequently, replicate them in other interest samples. Moreover, the influence of the semantic domain of common objects (living vs. non-living) on the ratings across dimensions requires further examination. Finally, socio-demographic variables (e.g., age, gender, education) should also be attended when producing normative studies of images of common objects.

A possible limitation of the current study was the constrain of the dimensions during the initial search, even though this specification allowed a better identification of potential studies. However, an additional hand-search enlarged the scope of the restrictions imposed by the key-words used in the Boolean search. Moreover, the choice of a qualitative approach may have circumscribed the significance of our findings to a limited context. Nevertheless, the present qualitative review may contribute as a guideline for further normative research. Finally, the use of h-index values as a journal quality measure does not exhaust all the available quality criteria and further comparisons across several existing measures might help in selecting the most representative one.

## Conclusion

Common objects are frequent and recognizable items that people encounter in their daily-lives. Therefore, they are recurrently used in several research and intervention domains. The normalization of this type of stimuli is imperative since they comprise specific characteristics and dimensions. Additionally, the use of poor-quality stimuli constitutes a constraint for scientific purposes, compromising the quality of the manipulations. The current review clearly indicates the need to produce further norms for realistic images of common objects in several dimensions across diverse linguistic and cultural contexts in a more systematic way as well as the necessity of advancing the normative field, namely in stimuli selection and standardization procedures. The main theoretical contribution of the current review is the endorsement of common objects as a broad category, with specific features, that deserves careful standardization for an optimal usage. Moreover, examining images of common objects as a distinct large category of images might emphasize their own relevance in the visual processing field and stimulate the production of new, robust and contextually-relevant datasets. From a practical perspective, the present review may inform future research designs (i.e., essential dimensions, methodological issues and findings, the selection of the stimuli) as well as help preventing the impact of undesirable confounding variables. Finally, normalizing materials with the purpose of safeguarding the quality of procedures—being they experimental or interventional—is an important research field on its own. Therefore, the current review emphasizes the normalization of visual stimulus as more than a procedure related to the researchers' everyday practices, but as a wide and rich research topic that should be acknowledged as such.

## Author Contributions

CS, MVG, and JCC contributed equally to design the procedures, to select the articles, and to draft the manuscript. CS executed the comprehensive search identification and selected all the relevant information for this review. MVG and JCC supervised and coordinated the whole process of producing this work and provided theoretical comments on the subject based on their expertise. All authors contributed to the article and approved the submitted version.

## Conflict of Interest

The authors declare that the research was conducted in the absence of any commercial or financial relationships that could be construed as a potential conflict of interest.

## Footnotes

^1^These dimensions were chosen based on a multifactorial perspective of visual processing, in which visuo-perceptive, affective and semantic components contribute to perception and visual recognition of everyday items (see Kensinger and Schacter, 2006; Brady et al., 2008; Konkle et al., 2010). Moreover, they constitute recurrent assessed variables from affective, semantic and perceptive domains.

^2^Studies that did not present correlations between dimensions were not considered in this analysis. For Berman et al. (1989), Cycowicz et al. (1997), and Pompéia et al. (2001) only the correlations obtained with adult samples are reported. In Bonin et al. (2013), Visual complexity was reported using objective measures. For studies reporting common objects as one category in contrast with other distinct type of stimuli (i.e., verb images) only results for common objects were included. See Supplementary Material S4, Table 2, for the entire list of extracted results across studies.

^3^Picture-name agreement refers to the agreement between name and image, with both features presented together (see Snodgrass and Vanderwart, 1980). Image agreement refers to the degree in which the visual representation of the stimuli fits well with the participant's previous mental image about the concept. Image agreement is considered very similar and as relevant as picture-name agreement, but the latter seems to involve less abstraction (see Snodgrass and Vanderwart, 1980 for details about both dimensions).
